# Trait-level predictors of human performance outcomes in personnel engaged in stressful laboratory and field tasks

**DOI:** 10.3389/fpsyg.2024.1449200

**Published:** 2024-09-09

**Authors:** Tad T. Brunyé, Sara Anne Goring, Julie A. Cantelon, Marianna D. Eddy, Seth Elkin-Frankston, Wade R. Elmore, Grace E. Giles, Clifford L. Hancock, Shoaib Bin Masud, James McIntyre, Kari L. McKenzie, K. Blake Mitchell, Meghan P. O’Donovan, Kenneth Racicot, John W. Ramsay

**Affiliations:** ^1^U.S. Army DEVCOM Soldier Center, Natick, MA, United States; ^2^Center for Applied Brain and Cognitive Sciences, Tufts University, Medford, MA, United States; ^3^Department of Electrical and Computer Engineering, Tufts University, Medford, MA, United States

**Keywords:** performance, prediction, traits, machine learning, military perception, memory, decision making, emotion

## Abstract

**Introduction:**

Personnel performance under stress hinges on various factors, including individual traits, training, context, mental and physiological states, and task demands. This study explored the link between the traits of military personnel and their performance outcomes in five domains: move, shoot, communicate, navigate, and sustain.

**Methods:**

A total of 387 U.S. Army soldiers participated in this study, undergoing trait assessments covering physical, cognitive, social–emotional, demographic/lifestyle, and health domains. Performance was measured through lab and field events assessing a broad range of individual and team-level skills under conditions demanding resilience to acute cognitive and physical stress exposure. Analysis used feature selection and elastic net regression.

**Results:**

Analyses revealed complex associations between traits and performance, with physical, cognitive, health-related, social–emotional, and lifestyle traits playing roles in guiding and constraining performance. Measures of resilience, emotion regulation, grit, and mindfulness were identified as relevant predictors of several performance-related outcomes.

**Discussion:**

Results carry implications for the selection, training, and operational effectiveness of personnel in high-stakes occupations including military and first response. Further research is necessary to explore the mechanisms underlying these associations and inform targeted interventions to boost personnel effectiveness.

## 1 Introduction

Human performance is inherently variable within and between individuals and across contexts, making it very challenging to predict (Newell, 1993; Smith et al., 2014). Relatively invariant individual traits across the physical, social–emotional, cognitive, demographic/lifestyle, and health domains may help account for inter-individual performance variability (Motowidlo et al., 1997; Muhs et al., 2018). However, while one or more trait-level variables might predict performance on a specific task executed in a specific context, those results may not generalize to performance in a novel context or with a modified task; indeed, the heterogeneity of performance contexts and tasks can make it difficult to generalize results between studies (Araújo et al., 2007; Salmon, 2020). Herein, our intent was to identify a reduced set of trait-level assessments (i.e., tasks, questionnaires) and measures (i.e., quantitative measures) that together can predict coarse performance constructs broadly applicable to Soldier performance across settings that demand resilience to acute stress.

### 1.1 Trait-level predictors of human performance

Human performance is a challenging and nebulous concept, with many different definitions proposed over the past several decades (Table 1). Definitions of human performance vary dramatically in level of analysis (e.g., individual versus group) and contextual specificity versus generality. They also vary regarding whether the processes underlying behavior (e.g., perception, emotion), behavior itself (i.e., observable outputs), and/or the results of behavior (e.g., winning a competition) are considered. In military training and operations, performance tends to be very outcome oriented, including assessments of task completion, efficiency and accuracy, and meeting or exceeding a superior’s intent. Performing tasks at an adequate level is continuously guided by mental and physical processes including attention, perception, memory, decision making, emotion, reasoning, agility, strength, endurance, flexibility, and balance.

**Table 1:** Extant definitions of *human performance* from the military and other related domains.

Citation	Definition	Context
Brimley et al. (2013)	*… a person’s physical, cognitive and social–emotional functions.*	Military
Travis and Brown (2023)	*… the successful completion of a specified task within an available performance capacity that meets or exceeds the mission demands*	Military
Naval Air Warfare Center (2021)	*… the range of perceptions, decisions, and actions that an individual or team carries out in the context of performing a task.*	Military
Britt et al. (2006)	*…how military personnel think, react, and behave in military operations.*	Military
Giles et al. (2023)	*…the extent to which individuals dynamically think, react, and behave relative to an established standard during training and operations.*	Military
EUROCONTROL/FAA Action Plan 15 Safety (2010)	*… the performance of jobs, tasks, and activities by operational personnel – individually and together.*	Air traffic control
Rothwell et al. (2012)	*… outcomes, results, and accomplishments achieved by a person, group, or organization.*	Organizational behavior
Fowler et al. (2019)	*… the act of performing and achieving a task, as distinct from performance shaping factors such as fatigue, task load, health or wellbeing, that can impact the task performance.*	Organizational behavior
Arra et al. (2023)	*… a worker’s capacity to meet the demands of a task.*	Organizational behavior
Quante et al. (2021)	*… the potential of a person to successfully perform a task.*	Automation and Robotics
Khripunov (2023)	*… actual behavior and the results of people’s actions, as opposed to an ideal or abstract view of what they are supposed to do.*	Human factors and ergonomics
Parush (2015)	*… perceptual, cognitive, emotional, and physical processes and behaviors.*	Human factors and ergonomics
Gawron (2000)	*… the accomplishment of a task by a human operator.*	Human factors and ergonomics
Reason (1990)	*… behavior plus results (P = B + R).*	Human factors and ergonomics
Hockey (1997)	*… the effectiveness or skill to accomplish goals through operations associated with human behavior.*	Human factors and ergonomics
Driskell and Salas (2013)	… *people doing things – performing a task, carrying out a procedure, solving a problem, or doing some type of work or activity.*	Human factors and ergonomics

The ability to predict human performance represents a formidable challenge for many occupational domains, with implications for personnel selection, training, and assessment. Performance prediction can be used to identify personnel who are likely to excel at particular occupational tasks, track the effectiveness of training trajectories, identify individualized performance optimization and enhancement techniques, optimize human-machine interaction, prevent injury and burnout, reduce the risk of stress disorders, and increase retention (Kaplan, 1965; Bartone et al., 2008; Campbell and Knapp, 2013; Farina et al., 2019; Brunyé et al., 2020; Samuelson et al., 2020). Herein, our analyses are restricted to the consideration of how traits predict performance. Traits are relatively invariant and enduring characteristics of individuals that differentiate them from others and have causal effects on performance (Steyer et al., 2015). While traits can indeed change over time due to experiences and training, we contrast traits with *states*, which are relatively transient and situationally dependent; states are not considered herein. In the present work, we conceptualize traits as falling into five general domains: physical, social–emotional, cognitive, demographic, and health-related.

#### 1.1.1 Physical traits

Physical traits are both structural and functional, such as body dimensions and composition, and strength and endurance (Casadei and Kiel, 2023). Not all physical traits are constant; indeed, some physical traits are highly invariant (e.g., height) over time whereas others might change slowly over the course of months or years (e.g., body mass). Physical traits have been linked to a variety of performance measures in both the physical and cognitive domains. For example, cardiovascular fitness is associated with cognitive function, functional brain connectivity, lower rates of mental health disorders, and stress reactivity (Belsky et al., 2015; Kronman et al., 2020; Schilling et al., 2020; Neumann et al., 2022). The precise nature of these relationships, including their strength and directionality, remains to be determined. The present study assessed several physical traits in military personnel, including aerobic and anaerobic endurance, agility and flexibility, body dimensions, activity level, power, and movement quality. Measuring these traits involved administering questionnaires, body/anthropometric measurements, anaerobic fitness assessments, functional movement screening, and the Army Combat Fitness Test (ACFT).

#### 1.1.2 Social–emotional traits

Social–emotional traits generally include those associated with relational and prosocial skills, and competence in recognizing and regulating emotions (Humphrey et al., 2011). While many social–emotional traits are likely relatively static (e.g., extraversion) over time, some might change through experiences and training (e.g., resilience). Social–emotional traits include relationship building and social problem-solving skills (including cooperation, turn-taking, and listening skills), self- and social-awareness, and the ability to regulate emotions and behavior and maintain resilience under conditions of stress and adversity. Resilience to stress exposure is likely a critical factor for sustaining performance, particularly in military contexts. During both training and operations, soldiers are frequently exposed to high-stress situations that can impact their cognitive and physical functioning. Psychological and physiological manifestations of resilience allow individuals to maintain optimal performance levels despite these challenges (Chappelle et al., 2018; Flood and Keegan, 2022; Biggs et al., 2023; McClung et al., 2023), providing a capacity to recover quickly from stress, adapt to changing environments, and remain focused under pressure (Algoe and Fredrickson, 2011; Franklin et al., 2012; Pfau and Russo, 2015; Crane et al., 2019; Kalisch et al., 2024). Resilience is not only vital for completing tasks efficiently but also for making accurate decisions, managing resources effectively, and maintaining overall mission readiness. In the present study, all performance tasks were performed under laboratory or field conditions that were inherently stressful (mentally and/or physically), as detailed in the Methods; those with higher trait levels of resilience are more likely to sustain performance under such conditions. The present study assessed several social–emotional traits in military personnel, including several self-reported measures of mindfulness, resilience, emotion regulation and stress responsiveness, impulsivity, personality, motivation, and trait affect.

#### 1.1.3 Cognitive traits

Cognitive traits are defined as the structural and functional central and peripheral nervous system characteristics that allow individuals to acquire, retain, and flexibly use information (Shettleworth, 2009; Croston et al., 2015). While most cognitive traits are relatively static (e.g., risk taking propensity) over time, some may change over months or years due to experience and training (e.g., working memory capacity). People vary in their ability to process information, control attention and behavior, update memory, learn new materials, apply knowledge, reason and produce judgments, and make decisions (Lee and Webb, 2005; Der and Deary, 2006; Deary et al., 2010; Kanai and Rees, 2011; Boogert et al., 2018; Naug and Tait, 2021). Cognitive traits have been linked to various performance measures across civilian and military domains including air traffic control skills, academic success, second language learning, multitasking, motor sequence learning, and resistance to misinformation (Ackerman, 1992; Bo and Seidler, 2009; Linck et al., 2009; Poole and Kane, 2009; Rabin et al., 2011; McVay and Kane, 2012; Wingo et al., 2013; Kapa and Colombo, 2014; Pollard and Courage, 2017; Robison and Unsworth, 2017; Brydges et al., 2018; Hanson et al., 2021). The present study assessed several cognitive traits in military personnel, including attention, mental flexibility, response inhibition, risk taking, working memory, mental rotation, and spatial perspective switching.

#### 1.1.4 Demographics and lifestyle

Demographics & Lifestyle related features are also highly heterogeneous across individuals; many are relatively static over time (e.g., handedness) whereas others change over the course of months or years (e.g., video game experience, exercise frequency). For example, military personnel vary in age, gender, handedness, race and ethnicity, education, and military experience and occupational specialization. They also vary in lifestyle choices such as whether they exercise, consume tobacco or alcohol, play video games, read, use social media, and engage in risky behavior. Both demographics and lifestyle choices have been previously linked to cognitive and physical performance. For example, information processing speed generally declines with age, inconsistent handedness predicts memory performance and cognitive flexibility, regular aerobic exercise increases executive function, chronic tobacco and alcohol use are associated with cognitive decrements, long-term dietary preferences and habits of military personnel are associated with physical performance during training, and video game experience predicts performance on military change detection, combat identification, and unmanned aerial vehicle control tasks (Hertzog, 1991; Ceballos, 2006; Durlach et al., 2009; Guiney and Machado, 2013; Prichard et al., 2013; Keebler et al., 2014; Lin et al., 2015). To capture some of these features, the present study administered questionnaires to assess a wide range of demographic and lifestyle variables.

#### 1.1.5 Health-related traits

Health-related traits generally include health-related behaviors such as sleep patterns and nutrition and eating, and health-related physiological status such as gut microbial community, and blood-based nutrient levels. Health-related traits, while invariant relative to acute health states, are likely to change over time through behavior or exogenous influences; for example, sleep habits and patterns of injury tend to shift during military training. In general, health-related traits have been related to cognitive and physical performance, with links to cognitive and physical performance and academic success in both civilians and military personnel (Reilly and Edwards, 2007; Valladares et al., 2016; Sellaro and Colzato, 2017; Spencer et al., 2017; Grandou et al., 2019; Craven et al., 2022; Petrofsky et al., 2022). The present study included blood draws, vision and hearing tests, and several questionnaires to probe enduring patterns of injury, sleep, and nutrition.

### 1.2 Human performance

To quantify performance, the present research focuses on basic warrior skills outlined in the Soldier’s Manual of Common Tasks (Department of the Army, 2009). The manual emphasizes several core competencies related to moving, shooting, communicating, navigating, and sustaining performance. In the present analyses, these competency domains were leveraged as *a priori* constructs to categorize measures that were collected across several study-related events (to be detailed in the Method section).

In military contexts and other high-stakes domains, the ability to sustain performance under conditions of mental and physical stress is critical to success. In many cases, including Special Operations Aviation Unit (SOAR) training, Ranger School, and Survive, Evade, Resist, and Escape (SERE) School training, military personnel are exposed to extreme and enduring stressors that affect training and are intentionally designed to mimic the realities of military operations (Burke and Dyer, 1984; Lieberman et al., 2005, 2016; Nindl et al., 2018; Vartanian et al., 2018). Acute stress exposures trigger a cascade of neuroendocrine and immune responses that begin with sympathetic-adrenal-medullary (SAM) responses and the release of catecholamines, and continue with activation of the hypothalamic–pituitary–adrenal (HPA) axis and the release of glucocorticoids (Christensen, 1991; Sapolsky et al., 2000; Vedhara et al., 2000; Schommer et al., 2003; Smith and Vale, 2006; Lee et al., 2012; Gagnon and Wagner, 2016; Cain and Cidlowski, 2017). Together, these stress systems produce diverse central and peripheral nervous system effects and modulate the musculoskeletal, cardiovascular, gastrointestinal, and immune systems. While mild to moderate stressors are often considered adaptive over the short term, intense and prolonged stress exposures are generally detrimental to performance. For example, exposure to intense acute stressors is associated with diminished cognitive and physical functions including decision-making, executive function, working memory, and visuomotor control and coordination (Charmandari et al., 2005; Schwabe et al., 2012; Starcke and Brand, 2012; Morey et al., 2015; Gagnon and Wagner, 2016; Shields et al., 2016; Martin et al., 2019).

In the present study, Soldiers completed a comprehensive baselining activity to quantify their traits; they then participated in one or more study-related events that were designed to induce acute stress in laboratory and field environments. For example, the study-related events induced stress through threat of shock, live-fire (i.e., using live ammunition) exercises, time pressure, load carriage, and team-level fast-paced movements through complex terrain while assaulting enemy positions. Importantly, each study event also embedded performance measures to quantify both cognitive and physical performance; broadly, these measures can be categorized into the military-relevant domains of move, shoot, communicate, navigate, and sustain.

#### 1.2.1 Move

A critical element of survival, Soldiers must be able to move effectively as a member of a team, under direct fire, and over, through, or around obstacles. Effective movement allows individuals and teams to avoid enemy targeting, leverage cover and concealment options, maintain visibility of enemy locations, and assume effective firing positions (Department of the Army, 2009). In the present study, we identified four measures of movement effectiveness related to performance on an anaerobic sprint test, aerobic capacity test, dynamic marksmanship test, and tactical movement in small unit formations.

#### 1.2.2 Shoot

A critical element of infantry performance is the ability to effectively maintain and employ a weapon, most commonly the M16 rifle or M4 carbine. Effective shooting involves zeroing a weapon and engaging stationary or moving targets at varied ranges, and meeting or exceeding the standard of hitting 60% or more of targets within an assigned sector (Department of the Army, 2009). In the present study, we identified 12 measures of shooting performance, considering both fundamental (e.g., weapon stabilization, engagement close to center of mass) and relatively operational (e.g., effectively deciding to shoot or not shoot, prioritizing targets) measures from study events involving virtual reality, simulated marksmanship, and field live-fire shooting events.

#### 1.2.3 Communicate

Performing voice communications with or without the assistance of a radio is an important element of team coordinated performance. Effective communication relies upon both language production and comprehension, and the ability to convey simple or complex concepts while following established standards (e.g., prowords, call signs, answering sequences) and using correct pronunciation and grammar (Department of the Army, 2009). In the present study, we identified three measures of communication performance related to speech accuracy and the exchange of information within small units (i.e., fire team and squad).

#### 1.2.4 Navigate

While small-scale navigation is reliant upon individual and team mobility, for example between cover and concealment options, large-scale navigation is also reliant upon spatial awareness of location and orientation of the self, relative to proximal and distal objectives. Effective navigation allows individuals and teams to move with efficiency and effectiveness between proximal and distal objectives while relying on perception, memory, and supporting devices (e.g., compass, map) (Department of the Army, 2009). In the present study, we identified a navigation-related measure that assesses the ability to orient oneself toward distal (and imperceptible) waypoints in large-scale space using memory and a compass.

#### 1.2.5 Sustain

A critical element of Soldier performance is the ability to effectively balance task-related demands and sustain physiological readiness throughout the duration of an exercise. Most related to the *survive* warrior skill (Department of the Army, 2009), effectively sustaining readiness over extended scenarios affords continuous movement, effective decision making and marksmanship, communication, and navigation through complex terrain. To quantify the ability to sustain physiological and neuromuscular readiness throughout the duration of an event, we identified eight performance measures examining the ability to maintain relatively low heart rates (relative to heart rate at VO2-max or age-predicted maximum heart rate) during challenging cognitive and physical work, maintain movement efficiency over the course of repeated bouts of physical exertion, and exert high levels of isometric torque on a dynamometer following strenuous physical work.

### 1.3 The present study

The present study examined whether traits of soldiers, including physical, social–emotional, cognitive, demographic/lifestyle, and health-related features, would predict performance measures across the domains of move, shoot, communicate, navigate, and sustain. A predictive model was built with 127 trait-level variables which were used as regressors to predict performance in military personnel. Note that we did not apply dimensionality reduction to these variables in order to retain traceability to the original assessment tools and their respective measures; indeed, one intent of this research was to precisely identify a reduced subset of assessments and measures that can be administered in the future to reliably predict performance. Dimensionality reduction of predictor variables, including principal components analysis, would not afford such traceability as predictors would be abstracted away from the original assessments.

To quantify performance, we used a breadth-first approach that aggregated data across a series of study events that took place in laboratory and field contexts, and with individuals and small teams. For each individual participant, we derived a total of five scores, one for each *a priori* performance domain: move, shoot, communicate, navigate, and sustain. These scores were used as outcomes in each of five models. This modeling approach allowed us to identify critical subsets of trait-level predictors (i.e., quantitative measures derived from trait assessments) that account for performance variation in each of the five domains while increasing probable generalizability of our findings across contexts and tasks.

## 2 Methods

### 2.1 Participants

A total of 387 enlisted U.S. Army soldiers (315 male, 4 female) participated voluntarily in a baselining event (when traits were measured) and then at least one of several study events. Military occupational specialties were predominantly infantry with the addition of some combat engineers and cavalry scouts. Demographic details of the sample are included in Table 2.

**Table 2:** Demographic details of the participant sample, including mean (and standard deviation) or participant counts.

Variable	Mean (SD) or counts
Military occupational specialty	
Infantry (11B or 11C)	*N* = 356
Combat engineer (12B)	*N* = 26
Cavalry scout (19D)	*N* = 5
Age (years)	23.3 (3.6)
Height (inches)	69.3 (4.2)
Weight (pounds)	179.8 (25)
Time in military (years)	2.8 (2.4)
Number of Deployments	0.4 (0.8)
Education	
Some high school completed	*N* = 4
High school degree or GED	*N* = 235
Some college completed	*N* = 107
Associate degree completed	*N* = 20
Bachelor’s degree completed	*N* = 21
Race and ethnicity	
American Indian or Alaskan Native	*N* = 8
Asian	*N* = 19
Black of African American	*N* = 22
Native Hawaiian or Other Pacific Islander	*N* = 6
White	*N* = 242
Hispanic or latino	*N* = 82

### 2.2 Study events and performance measures

Data were collected across multiple study events: one involving the collection of 127 trait-related variables (hereafter referred to as *features;* detailed in Table 3), and six involving the collection of performance-related variables (hereafter referred to as *outcomes*).

**Table 3:** The baseline assessments and features included in the analyses, including their domain, assessment used, features, and descriptive statistics.

Domain	Assessment	Feature	Mean, standard deviation
Physical	Range of motion test (ROM)	Crossbody reach while sitting	138.60, 44.23
		Standing forward extended reach	986.48, 59.56
		Standing overhead fingertip reach	2264.99, 99.01
		Standing trunk flexion	341.11, 92.10
		Thoraciclumbar (TL) spine rotation	129.85, 21.18
	Agility T-test	Trial time	12.05, 0.80
	Sit and reach test	Record trial	29.13, 8.23
	Functional movement screening (FMS)	Total score	509.30, 42.11
	Army combat fitness test (ACFT)	2-mile Run Time	80.29, 12.68
		Hand-release Push-up Total	85.20, 8.56
		Leg Tuck Total	84.54, 17.36
		Maximum deadlift total	86.62, 10.53
		Sprint-drag-carry total	89.31, 9.46
		Standing power throw total	81.93, 9.93
		Total score	509.30, 42.11
	Running anaerobic sprint test (RAST)	Anaerobic capacity	2512.86, 605.72
		Fatigue index	6.06, 3.20
	Seated power throw (SPT)	Maximum distance	595.85, 86.48
	International physical activity questionnaire (IPAQ)	Minutes/week of moderate MET	3703.74, 4723.01
		Total physical activity MET minutes/week	15127.66, 13635.77
		Vigorous activity MET minutes/week	8008.13, 7589.23
	Strength testing	Grip strength	49.40, 12.60
		Hip abduction	299.77, 96.44
		Hip adduction	163.58, 59.72
		Hip flexion	268.88, 94.40
		Knee extension	682.82, 191.90
		Knee flexion	292.71, 113.48
		Lumbar extension	700.18, 308.43
		Lumbar flexion	287.38, 113.02
		Pinch strength	10.05, 2.36
Social–Emotional	Five facet mindfulness questionnaire (FFMQ)	Act with awareness	27.32, 5.31
		Description	26.57, 5.17
		Observation	26.15, 5.31
		Non-judgmental	27.58, 5.83
		Non-reactivity	23.00, 3.95
	Positive and negative affect scale (PANAS)	Negative affect	17.31, 6.04
		Positive affect	34.46, 7.08
	Patient health questionnaire (PHQ)	Total score	4.77, 5.02
	Emotion regulation questionnaire (ERQ)	Cognitive reappraisal	29.38, 6.02
		Expressive suppression	17.98, 4.31
	Multi-family emotion regulation questionnaire (MFERQ)	Attentional deployment – distraction	18.49, 3.68
		Attentional deployment – rumination	15.40, 3.96
		Cognitive change	18.69, 3.97
		Mindfulness	16.86, 3.18		
Response modulation	16.56, 4.14				
Situation modification	19.59, 4.08				
Situation selection	18.85, 3.87			
Barratt impulsiveness scale (BIS)	Total impulsiveness score	63.62, 9.76		
Behavioral activation inhibition scale (BAIS)	Behavior activation score	23.13, 4.28		
	Behavior inhibition score	17.31, 3.48		
Big five personality inventory (BFPI)	Agreeableness	3.61, 0.78		
	Conscientiousness	3.77, 0.71		
	Extroversion	3.19, 0.91		
	Neuroticism	2.30, 0.78		
	Openness	3.35, 0.70		
Grit scale	Grit score	3.53, 0.52		
Connor-davidson resilience scale	Total score	76.28, 13.51		
State–trait anxiety index (STAI)	Trait anxiety	41.73, 6.90
Cognitive	Attentional blink task (ABT)	Blink magnitude	−0.07, 1.61
	Speed of processing task (SPT)	Reaction time	285.50, 100.44
	Visual search task (VST)	Reaction time slope	46.73, 27.63
	Task switching task (TST)	Switch cost	150.98, 132.66
	Go/no-go task (GNG)	Commission error rate	0.11, 0.11
	Flanker task (FT)	Flanker effect magnitude	35.97, 28.45
	Stroop task (ST)	Stroop effect magnitude	167.80, 104.79
	Balloon analog risk task (BART)	Overall performance	2135.17, 605.99
	Money road map task (MRMT)	Switch cost	0.08, 0.11
	Spatial working memory task (SWMT)	Accuracy	0.73, 0.11
	N-back task (NBT)	Mean accuracy	0.88, 0.08
Demographic, lifestyle	Health and habits questionnaire	Age	22.98, 3.19
		Number of deployments	0.31, 0.51
		Number of children	1.56, 0.78
		Years in military	2.56, 1.96
Health	Skin calipers/fold	Percent body fat	12.44, 4.36
	Anthropometry	Body mass index	26.66, 3.52
	Hearing test	Left ear ability	2.52, 2.96
		Right ear ability	2.15, 3.16
	Block food frequency questionnaire (BFFQ)	Alcohol	19.54, 23.13
		Caffeine	132.20, 112.93
		Carbohydrates	291.82, 168.97
		Kilocalories	2715.50, 1451.07
		Protein	112.69, 65.98
		Sugar	132.54, 87.75
		Fat	110.34, 61.25
	Mindful eating behavior scale (MEBS)	Nutrition knowledge score	36.69, 12.16	
Venous blood draws	Albumin (ALB)	4.03, 0.25		
	Alkaline phosphatase (ALP)	77.66, 23.35		
	Alanine transaminase (ALT)	28.31, 23.94		
	Aspartame aminotransferase (AST)	30.26, 20.84		
	Basophils	0.10, 0.09		
	Blood urea nitrogen (BUN)	13.18, 3.05		
	Calcium (CA)	9.73, 0.25		
	Chloride (CL)	104.37, 2.19		
	Copper	96.28, 14.92		
	Creatinine (CRE)	1.08, 0.20		
	Eosinophils	0.21, 0.12		
	Ferritin	86.44, 56.46		
	Folate	14.67, 4.92		
	Glucose	92.44, 6.57		
	Hematocrit	46.95, 3.07		
	Hemoglobin (HGB)	15.59, 1.04		
	Iron (FE)	101.72, 32.35		
	Potassium (K)	4.25, 0.33		
	Lymphocytes	2.38, 0.65		
	Magnesium (Mg)	2.03, 0.12		
	Mean corpuscular hemoglobin (MCH)	30.08, 1.42		
	Mean corpuscular hemoglobin concentration (MCHC)	33.21, 0.76		
	Mean corpuscular volume (MCV)	90.56, 3.32		
	Monocytes	0.56, 0.18		
	Mean platelet volume (MPV)	7.57, 1.38		
	Sodium (Na)	138.97, 2.22		
	Neutrophils	3.48, 1.53		
	Platelet count	243.49, 49.25		
	Red blood cell count (RBC)	5.19, 0.38		
	Red cell distribution width (RDW)	11.74, 0.55		
	Total bilirubin (TBIL)	0.93, 0.37		
	Total CO_2_ (TCO)	29.58, 1.60		
	Total iron-binding capacity (TIBC)	326.70, 42.34		
	Total protein (TP)	7.19, 0.41		
	Transferrin	266.64, 36.17		
	Transferrin saturation	31.69, 10.76		
	Vitamin B12	409.08, 129.84		
	Vitamin D	27.44, 7.61		
	Zinc	101.13, 14.83		
Morningness-eveningness questionnaire	Morning/evening score	41.19, 7.55		
Pittsburgh sleep quality index (PSQI)	Global score	7.03, 3.20		
Vision test at eccentricity	Overall accuracy	0.75, 0.21

For the collection of trait-related features, all participants completed a series of questionnaires, cognitive and physical tasks, and venous blood draws over the course of a three-to-five-day period (hereafter referred to as *baselining*). Note that baselining was not performed under conditions of stress, and took place on a rolling and as-needed basis (i.e., when needed for study events, between dates 2/21–8/23) and prior to participation in any other study events. The time between baselining and study event participation was within 4–6 weeks. Full methodological details of the baselining events can be found in (Giles et al., 2023); note that the present sample size exceeds that of this prior study, which was preliminary in nature.

Following baselining, participants completed at least one of the study events detailed in Table 4; there were a total of 50 performance outcomes derived from these events, distributed across the five *a priori* constructs (move, shoot, communicate, navigate, sustain).

**Table 4:** The seven study events included in our analyses, along with their respective protocol numbers, data collection settings (laboratory versus field), task descriptions, and outcome measures and domain.

Study event, protocol number, setting, dates	Description	Outcome measure(s), and domain(s)
Prediction Study: Cognitive Arm (18–007), Laboratory Setting, Data collection: 3/21–11/21	Individual participants are exposed to an acute physical stressor (threat of torso shock) while attempting to perform difficult marksmanship, memory, and spatial orienting tasks in virtual reality (Brunyé and Giles, 2023). Stressors are threat of electric shock, time pressure, and decision uncertainty.	Spatial orienting accuracy (navigate), target elimination efficiency (shoot), discrimination during marksmanship decision making (shoot), heart rate variability (sustain).
Prediction Study: Physical Arm (17–002), Laboratory Setting, Data collection: 2/22–8/22	Individual participants complete a strenuous bout of physical exertion and load carriage on a treadmill while attempting to perform difficult marksmanship, memory, and communication tasks on a computer display (Giles et al., 2022). Stressors are physical exertion, time pressure, and decision uncertainty.	Speech accuracy (communicate), VO2 max test time to exhaustion (move), discrimination during marksmanship decision making (shoot), percent of VO2 max (sustain), heart rate as percent of heart rate at VO2 max (sustain).
Small Unit Performance Analytics (SUPRA; 20–001 & 18–003, STUDY00001542), Field Setting, Data collection: 3/21–8/23	Squad-sized teams of participants execute infantry battle drill 2A in a situational tactical exercise (STX) lane while overall unit performance is assessed (O’Donovan et al., 2023). Stressors are physical exertion, decision uncertainty, time pressure, and social evaluation.	Observer-controller (OC) scores for information exchange (communications) and violence of action (move), time to kill first opposing force member (shoot), percent of max heart rate (sustain).
Repeated Bouts of Physical Stress (REPPS: 20–005), Laboratory Setting, Data collection: 4/21–5/23	Individual participants complete a multi-day simulated mission involving repeated ruck marches, accelerated movements, and scenarios involving react to contact battle drill (O’Fallon et al., 2022). Stressors are physical exertion, physical encumbrance, time pressure, and decision uncertainty.	Maintained isometric torque on dynamometer from pre- to post-task (sustain).
Tactical Stress Marksmanship Assessment (TSMA: 20–008), Field Setting, Data collection: 3/21–4/23	Individual participants complete running anaerobic sprint tests accompanied by live-fire marksmanship assessments while unloaded or carrying (~30 kg) loads (Cantelon et al., 2023). Stressors are physical exertion, physical encumbrance, time pressure, and threat of serious injury.	Time to complete running anaerobic sprint test (move), points during marksmanship course (shoot), marksmanship decision score (shoot), percent of max heart rate (sustain), maintained speed on successive bouts (sustain).
Individual Shooting Scenario (ISS: 20–005, 20–001, 18–003), Field Setting, Data collection: 6/21–8/23	Individual participants execute a scenario involving movement to a shooting position and engaging targets in a simulated marksmanship task (Brown et al., 2022a). Stressors are physical exertion (sprints), time pressure, and decision uncertainty.	Mobility score (move), fundamental marksmanship score (shoot), operational marksmanship score (shoot), overall lethality (shoot), weapon stability (shoot), marksmanship accuracy (shoot).
Team Shooting Scenario (TSS: 18–003), Field Setting, Data collection: 6/21–8/23	Fireteam-sized teams of participants execute a scenario involving an escalating-difficulty simulated marksmanship task requiring communication and coordinated responses (Brown et al., 2022b). Stressors are time pressure, decision uncertainty, and social evaluation.	Total communication score (communicate), marksmanship accuracy (shoot), percent of targets engaged (shoot).

The baselining and study events received ethical approvals through the institutional review board(s) (IRB) at the United States Army DEVCOM Armaments Center, Walter Reed Army Institute of Research (WRAIR) IRB, and/or Tufts University (IRB protocol numbers included in Table 4).

### 2.3 Data processing

Baseline data were processed and summarized as previously outlined (Giles et al., 2023), resulting in a total of 127 trait-level features for use in our analyses. These features are listed in Table 3, and were standardized prior to inclusion in analyses. As detailed in Table 4, a total of 28 performance outcomes were available for analysis from the study events; each was categorized into a single performance outcome domain (move, shoot, communicate, navigate, sustain) based on the results of consensus meetings among authors.

Because all participants were involved in the baselining event but then variably involved in the subsequent study events, a method was needed to normalize and aggregate data across study events and their respective performance outcomes. To do so, each performance outcome was standardized to result in scaled data with a mean of 0 and standard deviation of 1. The additive inverse of the standardized score was used for any outcomes with higher values indicating poorer performance (for example, distance from center of mass or time to completion). In this manner, higher standardized scores within each measure consistently indicated higher performance. Then, within each *a priori* performance domain, we computed a mean standardized outcome score for each participant by averaging across each domain’s standardized outcome with each being equally weighted. The result of this process was a single outcome for each participant and performance domain, affording a regression-based analysis.

### 2.4 Data analysis

Data were analyzed in two phases: evaluation/elimination of features based on regression diagnostics and inter-rater-reliable priors, followed by feature-selection and model fitting using elastic net regularization techniques (Zou and Hastie, 2005). Note that because participants were variably involved in study events, each of the five composite performance outcomes had a different number of participants included in analysis (see Table 5).For regression diagnostics, the features were evaluated for collinearity in addition to assessing their individual associations with each of the five outcomes (Meuleman et al., 2014; Fox, 2019). Features that were correlated with other features above a threshold (≥ 0.80), that also produced variance inflation factors (VIFs) over 5 when included in the same model, were removed from the outcome subset (Belsley et al., 1980; Chatterjee and Simonoff, 2013; Dunn and Smyth, 2018). Additional features were also removed for lacking meaningful associations with the outcome if also deemed by priors as “unlikely” or “very unlikely” to be feature-selected for that specific outcome. Priors were based on ratings from a panel of five subject matter experts (cognitive, biomedical, and biomechanical science) who were asked to characterize the relationship between each feature and outcome. The subjective ratings were based on the relevance of each feature in predicting each of the five outcomes (move, shoot, communicate, navigate, sustain), and responses were provided using a five-point Likert scale ranging from Very Unlikely (1) to Very Likely (5). After elimination, the remaining features were distributed across the five outcomes as detailed in Table 5. Overall, the combination of regression diagnostics and input from specified priors allowed us to eliminate features from each model and facilitate a more parsimonious solution.

**Table 5:** The number of features (by feature category: physical, social–emotional, cognitive, demographic, health) included in each of the five models, at each of the phases (pre-regression diagnostics, post-regression diagnostics, post-feature selection).

Study phase	Performance outcome (N)	Physical features	Social–emotional features	Cognitive features	Demo-graphic features	Health features
Pre-regression diagnostics	Move (128)	30	28	11	4	54
	Shoot (156)	30	28	11	4	54
	Communicate (124)	30	28	11	4	54
	Navigate (83)	30	28	11	4	54
	Sustain (151)	30	28	11	4	54
Post-regression diagnostics	Move (128)	28	11	3	4	5
	Shoot (156)	28	22	11	4	13
	Communicate (124)	28	28	11	4	21
	Navigate (83)	28	28	11	4	16
	Sustain (151)	28	28	11	4	29
Post-feature selection	Move (128)	11	4	5	2	2
	Shoot (156)	28	22	11	4	13
	Communicate (124)	17	3	4	2	13
	Navigate (83)	2	2	2	0	3
	Sustain (151)	6	3	2	0	5

Number of participants (N) included in each phase is also included.

Due to the large number of features remaining after the previous step and the associated risk of overfitting, elastic net regression was employed as a model regularization technique and feature selection method (Zou and Hastie, 2005). Elastic net is a machine-learning based algorithm that combines the L1 and L2 penalty features of lasso and ridge regression regularization methods to favor more parsimonious model solutions. The elastic net regression fitting method is particularly useful for instances in which there are many features relative to the number of participants (sample size), and for models containing groups of correlated variables (Friedman et al., 2010). These issues were relevant to the current data, despite removal of some highly correlated features in the preliminary diagnostics stage. Additionally, elastic net techniques offer a combination of feature selection and coefficient shrinkage to identify the most relevant features and reduce the other feature coefficients toward zero. Feature selection on its own would risk an imbalance of variance in the models, leading to an overly complex, sample-specific set of results. By also introducing some bias to the model via shrinkage, it is more consistent with the goal of establishing a subset of features that are generalizable to the testing of future samples.

Analyses were conducted using R Statistical Software (v2023.06.1 + 524; R Core Team) with the caret package (v6.0–94) (Kuhn, 2008). Five linear regression models were fitted, one for each outcome, using elastic net regularization methods optimized by 10-fold cross-validation. A 100-item parameter grid was used to find the ideal model combination for a range of mixing parameter values (α, 0–1) and tuning parameter values (λ, 0–1) to determine the best fit to each outcome subset. The final models were selected based on the parameter combination that minimized the root-mean-squared-error (RMSE), while maximizing the proportion of variance explained by the model (R^2^). The regression coefficients generated from the final models are reported in Table 6, and model parameters and fit indices are detailed in the respective results sections for each composite outcome.

**Table 6:** Results of the elastic net regression process including results for each composite outcome, the selected features, and the feature-specific regression coefficients.

Move outcome (24 features, *R*^2^ = 0.16)	Coefficient
Health, blood magnesium	0.229
Cognitive, useful field of view accuracy	0.181
Lifestyle, number of deployments	−0.165
Physical, agility trial time	−0.052
Lifestyle, number of years in military	−0.014
Physical, ACFT hand release push-up total	−0.006
Social/emotional, PHQ total score	−0.004
Physical, flexibility maximum	0.004
Cognitive, visual search task RT slope	0.003
Social/emotional, behavior activation system	0.003
Health, alcohol consumption	0.002
Social/emotional, emotion regulation - situation modification	−0.002
Physical, ACFT maximum deadlift	0.001
Cognitive, flanker effect	0.001
Physical, average grip strength	0.001
Physical, average hip adduction	0.001
Physical, average standing forward extended reach	0.001
Cognitive, stroop effect	<0.001
Cognitive, behavior inhibition system	<0.001
Social/emotional, total resiliency	<0.001
Physical, ACFT leg tuck total	<0.001
Physical, average cross-body reach	<0.001
Physical, ACFT 2-mile run time	<0.001
Physical, moderate MET minutes per week	<0.001
Shoot outcome (78 features, *R*^2^ = 0.15)	Coefficient
Cognitive, spatial working memory accuracy	0.601
Cognitive, spatial perspective-taking accuracy cost	−0.456
Health, blood potassium	−0.369
Lifestyle, number of children	0.275
Cognitive, go/no-go inhibition commission error rate	0.192
Cognitive, N-back working memory accuracy	−0.070
Physical, agility trial time	−0.052
Social/emotional, grit score	−0.046
Social/emotional, emotion regulation - situation modification	−0.033
Social/emotional, mindfulness - acting with awareness	−0.030
Social/emotional, mindfulness	−0.029
Social/emotional, mindfulness - non-judging	0.027
Health, left ear hearing ability	0.025
Social/emotional, expressive suppression	0.021
Physical, flexibility total score	0.020
Physical, maximum flexibility	−0.020
Social/emotional, barrett impulsiveness scale total score	−0.019
Cognitive, attentional blink magnitude	−0.018
Social/emotional, emotional regulation cognitive change	0.017
Lifestyle, years in military	0.016
Health, body mass index	−0.015
Health, right ear hearing ability	−0.015
Lifestyle, age	−0.014
Health, nutrition knowledge score	0.014
Social/emotional, emotion regulation - situation selection	0.012
Social/emotional, behavior inhibition system	0.012
Social/emotional, negative affect	0.012
Lifestyle, number of deployments	−0.011
Social/emotional, emotion regulation - cognitive reappraisal	−0.011
Social/emotional, attentional deployment - rumination	0.009
Social/emotional, behavior activation system	−0.008
Physical, ACFT hand release push-up total	0.008
Physical, ACFT standing power throw total	0.007
Physical, average thoracic lumbar spine rotation	−0.005
Social/emotional, total affect	0.005
Social/emotional, attentional deployment - distraction	0.004
Physical, ACFT leg tuck total	−0.004
Physical, ACFT 2-mile run time	−0.004
Physical, ACFT maximum deadlift	−0.003
Physical, ACFT total score	0.003
Physical, average pinch strength	−0.003
Physical, running-based anaerobic sprint test fatigue index	0.002
Social/emotional, positive Affect	−0.002
Health, sugar consumption	−0.002
Physical, average standing trunk flexion	−0.002
Cognitive, visual search task RT slope	−0.002
Health, blood transferrin saturation	0.001
Physical, seated power throw maximum	0.001
Cognitive, stroop effect	−0.001
Physical, ACFT sprint drag carry total	−0.001
Health, carbohydrate consumption	0.001
Physical, average hip adduction	−0.001
Social/emotional, emotion regulation - response modulation	−0.001
Social/emotional, mindfulness - describe	0.001
Physical, average knee flexion	−0.001
Physical, average lumbar flexion	−0.001
Health, caffeine consumption	0.001
Physical, average knee extension	0.001
Health, blood transferrin	−0.001
Social/emotional, mindfulness - observe	0.001
Cognitive, speed of processing RT	−0.001
Social/emotional, resilience total	0.001
Physical, average hip flexion	0.001
Physical, average grip strength	<0.001
Cognitive, task switching cost	<0.001
Social/emotional, mindfulness – non-react	<0.001
Physical, average standing overhead fingertip reach	<0.001
Physical, average hip abduction	<0.001
Health, alcohol consumption	<0.001
Physical, anaerobic capacity	<0.001
Physical, average lumbar extension	<0.001
Health, protein consumption	<0.001
Physical, average cross-body reach	<0.001
Physical, average standing forward extended reach	<0.001
Health, KCal consumption	<0.001
Cognitive, flanker effect	<0.001
Cognitive, risk-taking task overall performance	<0.001
Physical, moderate MET minutes per week	<0.001
Communicate outcome (39 Features, *R*^2^ = 0.25)	
Health, blood basophils	−0.255
Health, blood potassium	−0.142
Cognitive, N-back working memory accuracy	−0.080
Social/emotional, conscientiousness score	−0.060
Health, blood hemoglobin	−0.040
Lifestyle, number of deployments	−0.033
Health, blood sodium	−0.030
Cognitive, spatial perspective-taking accuracy cost	−0.025
Physical, average pinch strength	0.022
Health, blood urea nitrogen	−0.021
Physical, agility trial time	−0.017
Social/emotional, expressive suppression	−0.015
Social/emotional, emotion regulation – response modulation	−0.013
Health, blood chloride	−0.011
Health, nutrition knowledge score	−0.008
Physical, fatigue index	0.008
Physical, ACFT sprint drag carry total	0.004
Physical, ACFT leg tuck total	0.003
Health, blood hematocrit	−0.003
Health, blood vitamin D25	0.002
Physical, average thoracic lumbar spine rotation	−0.002
Physical, flexibility total score	0.002
Health, percent body fat	−0.002
Physical, ACFT hand release push-up total	0.002
Physical, average hip adduction	0.001
Health, blood carbon dioxide	0.001
Cognitive, flanker effect	0.001
Health, blood zinc	0.001
Physical, ACFT total score	<0.001
Health, right ear hearing ability	<0.001
Physical, average standing overhead fingertip reach	<0.001
Physical, average lumbar flexion	<0.001
Physical, average lumbar extension	<0.001
Physical, anaerobic capacity	<0.001
Physical, average knee flexion	<0.001
Lifestyle, years in military	<0.001
Physical, ACFT standing power throw total	<0.001
Physical, moderate MET minutes per week	<0.001
Cognitive, attentional blink magnitude	<0.001
Navigate outcome (9 features, *R*^2^ = 0.39)	
Cognitive, spatial working memory accuracy	2.864
Cognitive, spatial perspective-taking accuracy cost	−1.396
Social/emotional, grit score	−0.030
Social/emotional, mindfulness – observe	0.008
Physical, average hip flexion	0.002
Health, nutrition knowledge score	0.001
Health, blood zinc	0.001
Health, caffeine consumption	<0.001
Physical, average knee extension	<0.001
Sustain outcome (16 Features, *R*^2^ = 0.12)	
Health, blood potassium	0.062
Social/emotional, neuroticism score	−0.028
Cognitive, attentional blink magnitude	0.023
Social/emotional, emotional regulation – mindfulness	−0.011
Health, blood folate	−0.004
Health, blood mean platelet volume	0.001
Social/emotional, mindfulness – non-react	−0.001
Cognitive, flanker effect	0.001
Health, blood copper	−0.001
Physical, average pinch strength	<0.001
Physical, ACFT leg tuck total	<0.001
Health, blood platelet count	<0.001
Physical, average standing trunk flexion	<0.001
Physical, average lumbar extension	<0.001
Physical, anaerobic capacity	<0.001
Physical, average standing overhead fingertip reach	<0.001

## 3 Results

Table 5 details the number of features remaining in each model after the feature selection process.

### 3.1 Move outcome

The final model (*α* = 0.10, λ = 0.46; *RMSE* = 0.68, *R^2^* = 0.16) selected 24 of the 54 features, accounting for 16% of the variance in the *move* outcome. The regression coefficients generated from the model are reported in Table 6; 11 of the features were related to physical traits (ACFT measures, agility, flexibility, strength, activity levels), 5 were related to cognitive traits (inhibitory control, useful field of view, visual search ability), 2 were related to health traits (alcohol consumption, blood magnesium), 4 were related to social/emotional traits (emotion regulation, resilience), and 2 were related to lifestyle traits (number of deployments, years in military).

### 3.2 Shoot outcome

The final model (*α* = 0.60, λ = 0.00; *RMSE* = 0.96, *R^2^* = 0.15) selected all 78 features, accounting for 15% of the variance in the *shoot* outcome. The regression coefficients generated from the model are reported in Table 6; 28 of the features were related to physical traits (ACFT measures, agility, flexibility, strength, speed, endurance, activity levels), 11 were related to cognitive traits (attention, inhibitory control, working memory, risk taking, spatial perspective taking, speed of processing, useful field of view, visual search ability), 13 were related to health traits (blood analytes, consumption patterns, hearing ability), 22 were related to social/emotional traits (impulsiveness, behavioral activation and inhibition, emotion regulation, grit, mindfulness, resilience), and 4 were related to lifestyle traits (age, number of children, number of deployments, years in military).

### 3.3 Communicate outcome

The final model (*α* = 0.01, λ = 1.00; *RMSE* = 0.99, *R^2^* = 0.25) selected 39 of the 92 features, accounting for 25% of the variance in the *communicate* outcome. The regression coefficients generated from the model are reported in Table 6; 17 of the features were related to physical traits (ACFT measures, agility, flexibility, endurance, activity levels), 4 were related to cognitive traits (attention, inhibitory control, working memory, spatial perspective taking), 13 were related to health traits (blood analytes, nutrition knowledge, percent body fat, hearing ability), 3 were related to social/emotional traits (conscientiousness, emotion regulation, expressive suppression), and 2 were related to lifestyle traits (number of deployments, years in military).

### 3.4 Navigate outcome

The final model (*α* = 0.90, λ = 0.18; RMSE = 0.85, R2 = 0.39) selected 9 of the 88 features, accounting for 39% of the variance in the *navigation* outcome. The regression coefficients generated from the model are reported in Table 6; 2 of the features were related to physical traits (flexibility), 2 were related to cognitive traits (spatial working memory, spatial perspective taking), 3 were related to health traits (blood zinc, nutrition knowledge, caffeine consumption), 2 were related to social/emotional traits (grit, mindfulness), and 0 were related to lifestyle traits.

### 3.5 Sustain outcome

The final model (*α* = 0.10, λ = 0.91; RMSE = 0.81, R2 = 0.12) selected 16 of the 101 features, accounting for 12% of the variance in the *sustain* outcome. The regression coefficients generated from the model are reported in Table 5; 6 of the features were related to physical traits (ACFT leg tuck, endurance, strength, flexibility), 2 were related to cognitive traits (attention, inhibitory control), 5 were related to health traits (blood analytes), 3 were related to social/emotional traits (emotion regulation, mindfulness, neuroticism), and 0 were related to lifestyle traits.

### 3.6 Outcome summary

Across the five outcomes, no single feature was selected in all five models. The most frequently selected features that were selected in at least 2 of the 5 models are included in Table 7. Overall, across models the most frequently selected features were physical (30 features), followed by Social/Emotional (29 features), Health (28 features), Cognitive (13 features), and Lifestyle (5 features). Interestingly, while none of the same features were selected across all five models, in general trait-level measures of grit, emotion regulation, resilience, and/or mindfulness held predictive value across many of the outcomes.

**Table 7:** The most frequently selected features across the five models, including the feature name and the models selecting the feature.

Feature	Models selecting feature
Cognitive, spatial perspective-taking accuracy cost	3: Move, Communicate, Sustain
Cognitive, flanker effect	3: Shoot, Communicate, Navigate
Health, blood potassium	3: Shoot, Communicate, Sustain
Health, nutrition knowledge score	3: Shoot, Communicate, Navigate
Lifestyle, number of deployments	3: Move, Shoot, Communicate
Physical, AFCT hand release push-up total	3: Move, Shoot, Communicate
Physical, agility trial time	3: Move, Shoot, Communicate
Physical: average hip adduction	3: Move, Shoot, Communicate
Cognitive, attentional blink magnitude	2: Shoot, Sustain
Cognitive, N-back working memory accuracy	2: Shoot, Communicate
Cognitive, spatial working memory accuracy	2: Shoot, Navigate
Cognitive, visual search task RT slope	2: Move, Shoot
Health, blood zinc	2: Communicate, Navigate
Physical, ACFT leg tuck total	2: Shoot, Communicate
Physical, ACFT maximum deadlift	2: Move, Shoot
Physical, ACFT sprint drag carry total	2: Shoot, Communicate
Physical, average hip flexion	2: Shoot, Navigate
Physical, average pinch strength	2: Shoot, Communicate
Physical, average thoracic lumbar spine rotation	2: Shoot, Communicate
Physical, flexibility total score	2: Shoot, Communicate
Social/emotional, behavior activation system	2: Move, Shoot
Social/emotional, emotion regulation - situation modification	2: Move, Shoot
Social/emotional, expressive suppression	2: Shoot, Communicate
Social/emotional, grit score	2: Shoot, Navigate

It is also important to point out that model error (as assessed via RMSE) was moderately high across all five models, suggesting that the model’s predictions are, on average, different from the actual observed values, indicating poor model accuracy. This often implies that the model is not capturing the underlying patterns in the data effectively, suggesting that different features (e.g., states) may prove valuable for complementing trait-based features.

## 4 Discussion

In this study, we investigated whether soldiers’ trait-level traits are associated with cognitive and physical performance outcomes across a range of laboratory and field tasks. Results indicated that traits measured across physical, social–emotional, cognitive, demographic/lifestyle, and health-related domains collectively contribute to explaining low-to-moderate variance in performance outcomes related to several domains critical for military occupational functioning: move, shoot, communicate, navigate, and sustain. The varied nature of features identified in each performance outcome underscores the multifaceted nature of human performance and highlights the importance of considering a broad array of individual traits when assessing, predicting, and optimizing performance.

When predicting the move domain, the model selected 24 diverse features but was able to only account for 16% of variance in the outcome, showing moderate model error (as indicated by RMSE values). Among the physical features, we found that several baseline measures of strength (e.g., maximum deadlift) and flexibility (e.g., maximum flexibility) were positively associated with performance outcomes; similarly, agility trial time (i.e., the time to complete the agility trial) was negatively associated with the move outcome. However, we also found a measure of power (i.e., ACFT hand release push-up total) was (unexpectedly) negatively associated with the move outcome. It is not entirely clear why this association arose; perhaps because move outcomes were derived primarily from tests of lower body movement (e.g., sprint tests, treadmill tests), upper body power is unrelated to, or even counterproductive to, their execution. That said, our findings generally align with previous research highlighting the importance of physical conditioning and readiness in military contexts, with an emphasis on strength, flexibility, and agility (Heinrich et al., 2012; Kraemer and Szivak, 2012; Lester et al., 2014; Smith et al., 2022).

When predicting the shoot domain, the model selected all available features but was only able to account for 15% of variance in the outcome, showing relatively high model error. Most prevalent among selected variables were physical traits, indicating the importance of power and endurance, speed, strength, agility, and flexibility. The strongest physical predictor was related to the amount of time it took to complete an agility trial, with those completing it faster also showing higher shoot outcomes; given that many of the shooting tasks examined herein involved acute bouts of physical exertion between shooting episodes (e.g., running between supported firing positions), this result is intuitive but also points to the importance of whole-body coordinated activity for attaining high marksmanship performance. Among the cognitive traits, the strongest features were related spatial cognitive processes, specifically the ability to temporarily maintain spatial information in working memory and transform spatial perspectives. The visuo-motor coordination involved in aiming a weapon and acquiring targets is inherently spatial, involving dynamic coordination of the body, head and eyes with the weapon’s intrinsic axes and variably sized and positioned external targets (Chung et al., 2004; Dudde et al., 2012; Palmer and Van Emmerik, 2020; Brown et al., 2022b). The ability to process, manipulate, and transform spatial information appears to critically underpin effective shooting performance, a possibility worthy of future investigation. This is particularly intriguing given the relative trainability of spatial skills and their apparently successful transfer to untrained tasks (Uttal et al., 2013). We also found that baseline performance on cognitive tasks examining the ability to effortfully control attention and switch between tasks was associated with shoot performance, supporting prior work demonstrating the role of executive functions in shoot/do not-shoot decisions (Biggs et al., 2015; Biggs, 2021; Biggs and Pettijohn, 2022). Among the health-related traits, blood levels of potassium were most strongly and negatively associated with shoot performance. High blood potassium levels (hyperkalemia) are associated with muscle weakness and fatigue (Makuch et al., 2019), which could play a role in the ability to effectively manage a weapon during strenuous physical activities. Finally, among lifestyle features, number of children emerged as strongly and positively related to shoot performance. While we are unaware of any previous research demonstrating this association, some studies suggest that parents evaluate themselves and their lives more positively than those without children, and they experience more positive emotions, gratification, and feelings of meaning and contribution (Umberson and Gove, 1989; White and Dolan, 2009; Nelson et al., 2013). Parents with more children might be especially adept at regulating their own emotions and those of others (Rutherford et al., 2015; Grolleman et al., 2023), and this may influence performance during stressful shooting events. Continuing research will explore these possible relationships and how they may moderate effects of parenthood on shooting performance.

When predicting the communicate domain, the model selected a high number of features (39) to account for a low-to-moderate amount of variance (25%) but with relatively high model error. The strongest features were related to blood analytes, working memory, conscientiousness, and number of deployments. For health-related blood analytes, we found that lower levels of basophils, potassium, hemoglobin, and sodium were related to higher communication performance. Basophils generally have an anti-inflammatory effect via histamine content, and blood potassium, sodium, and hemoglobin have been variably associated with cognitive function in clinical or aging populations (Jáuregui-Lobera, 2014; Cisternas et al., 2015; Kung et al., 2021; Gomes et al., 2023; Harner and Root, 2023; Suárez et al., 2024). Given the novelty of these health-related features for predicting communications outcomes, replication and extension will be important. In the cognitive domain, lower working memory accuracy on the n-back task was associated with higher communication outcomes. Some theories of working memory suggest that performance on working memory tasks is critically dependent upon executive function (Engle, 2002; Engle and Kane, 2003; McCabe et al., 2010); it could be the case that those with lower working memory capacity show lower inhibition levels during communication tasks, making them more likely to produce language that ultimately helps support team-oriented tasks.

When predicting the navigate domain, the model selected very few features but accounted for moderate amounts of variance (39%) with moderate model error. Selected features were predominantly cognitive in nature. The high variance accounted for in the model is likely because only a single performance measure was used to calculate the navigate outcome, derived from a single study; this may decrease the inter-measure variance associated with the composite performance outcome and facilitate prediction. However, it likely also reduces the generalizability of our results within this domain. Within the cognitive domain, working memory and perspective-taking were both positively associated with higher navigation performance. Specifically, higher spatial working memory scores and lower cost associated with switching perspectives on a spatial task, were both positively associated with this outcome. Visuo-spatial working memory is considered a fundamental process in successful performance on spatial cognition tasks including localization, orientation, and navigation (Garden et al., 2002; Baumann et al., 2011; Meneghetti et al., 2016, 2021), as are the attentional control and executive function skills required to transform spatial perspectives (Korthauer et al., 2017).

When predicting the sustain domain, the model selected only 16 features and only accounted for 12% of variance in the outcome, with moderate model error. The strongest association was a positive relationship between blood potassium and the ability to sustain speed or strength during acute bouts of physical exertion. Potassium plays a crucial role in various physiological processes, including muscle function, fluid balance, nerve transmission, and energy metabolism (Nielsen et al., 1986; Maughan et al., 1997; Sejersted and Sjøgaard, 2000; Demigné et al., 2004). Given the essential contribution of potassium to the proper functioning of muscle cells, low potassium levels, a condition known as hypokalemia, are associated with muscle weakness and fatigue. Alongside sodium, potassium also helps regulate fluid balance within cells and throughout the body, and maintaining adequate potassium levels can help prevent dehydration. Potassium is also involved in nerve transmission (e.g., between the brain and muscles) that supports coordinated movement and muscle contractions during exercise. Finally, potassium also plays a role in the conversion of carbohydrates into energy. It could be the case that those with higher basal levels of blood potassium may be better equipped to sustain adequate potassium levels to support exercise performance. Continuing research will be critical to assess the reliability and scope of such an effect, and identify its precise physiological mechanisms.

Interestingly, while no single feature was selected consistently across all five composite performance outcomes, there was some suggestion that features related to resilience were generally important for predicting several outcomes. For example, individual differences in emotion regulation were important for predicting move, shoot, communicate, and sustain. Individual differences in measures of resilience were important for predicting move and shoot outcomes, grit was important for predicting shoot and navigate outcomes, and mindfulness was important for predicting shoot, navigate, and sustain outcomes. Emotion regulation refers to the processes involved in managing and adaptively responding to emotionally salient experiences; when individuals with relatively high emotion regulation skills encounter adversity, they are better able to handle the adversity (maintain composure, reframe experiences, recover from setbacks) without becoming overwhelmed, a key protective factor in overall resilience (Troy and Mauss, 2011; Gross, 2013, 2015). Grit refers to a passion for and perseverance toward goals, which involves sustaining interest and effort in the face of challenges; those with higher grit are more likely to persist through challenges and continually striving toward goals, a core aspect of resilience (Duckworth et al., 2007; Caza et al., 2020; Datu, 2021). Mindfulness is also related to resilience because it allows individuals to maintain a balanced perspective in the face of stress, restrain from immediate reactions, and maintain a calm and composed mindset (Pidgeon and Keye, 2014). Indeed some research suggests that mindfulness-based stress reduction (MBSR) training improves resilience via increases in several aspects of mindfulness (e.g., decentering, acceptance) (Nila et al., 2016). The fact that numerous traits related to resilience were predictive of performance outcomes is compelling, suggesting that broad resilience-related traits are critical for sustaining real-world performance.

### 4.1 Implications for performance prediction, assessment, and training

Our findings have several implications for personnel selection, training, and operational practices. By identifying key trait-level predictors of performance outcomes, military organizations can tailor selection criteria, training programs, and support interventions to optimize individual and team performance. For instance, integrating targeted physical conditioning, cognitive training, and stress resilience programs into training curricula may enhance overall readiness and effectiveness in diverse operational contexts. Moreover, leveraging predictive models derived from measured traits can inform personnel assignment, task allocation, and performance optimization strategies, thereby enhancing mission success and personnel well-being.

A primary goal of this research was to identify a subset of traits that could be easily assessed in laboratory and field environments but prove helpful for predicting diverse performance outcomes relevant to military training and operations. We began with a set of 127 features that were measured by administering 40 diverse trait assessments (i.e., questionnaires and cognitive and physical tasks) and a venous blood draw to participants over the course of a multi-day baselining event. Through the modeling process, we were able to identify the most critical set of trait assessments to administer when attempting to predict each outcome. Specifically, to predict *move* there are only 16 assessments to administer, shoot has 34 assessments, communicate has 19 assessments, navigate has 9 assessments, and sustain has 10 assessments. In other words, we were able to reduce the number of assessments to be administered by 18–80% depending upon which outcome is to be predicted. For example, to predict the *navigate* outcome, scientists and practitioners can account for 39% of variance in this outcome by only administering the following 9 assessments: Block Food Frequency Questionnaire, Five Facet Mindfulness Questionnaire, Grit Scale, Hearing Tests, Military Eating Behavior Survey, Money Road Map Task, Spatial Working Memory Test, Strength Tests, and Venipuncture for Zinc. In this case, rather than requiring a multi-day baselining event to administer 41 assessments, this reduced baselining could likely take place over the course of 3–4 h. Other outcomes are more difficult to predict and require administering more assessments while accounting for relatively low outcome variance.

### 4.2 Limitations and future directions

Despite the valuable insights gained from this study, several limitations warrant consideration. First, the sample predominantly consisted of white male soldiers, limiting the generalizability of findings to relatively diverse military populations. Future research should aim to include more diverse samples to capture the full spectrum of trait-performance relationships across different demographic groups and military occupational specialties. Additionally, the study focused on trait-level variables and did not account for situational, contextual, or genetic factors that may influence performance outcomes. Future studies should explore the interactive effects of these variables on performance to provide a more comprehensive understanding of human performance dynamics in military settings; it is likely that the inclusion of more comprehensive characterizations of individuals will increase the variance accounted for in performance outcomes. It may also help reduce the relatively high error (measured as RMSE) in our models, which indicate that the models’ predictions are, on average, different from the actual observed values, suggesting relatively low model accuracy. Some of this is related to the inherent bias-variance trade-off, and the risk of producing an overly complex model due to the large number of features relative to observations. The regularization techniques used to avoid this outcome increase the bias of the model to produce a more generalizable result. However, this does decrease the sensitivity and predictive power to capture patterns in the current data. Some analytic approaches, including ensemble methods and decision trees, might assist in reducing model error and increase precision, but they may be less appropriate for high dimensional data due to the risk of overfitting.

Furthermore, some traits are more invariant than others; indeed, some traits will change through young adulthood (e.g., age, number of children) and some will remain relatively constant (e.g., personality, working memory capacity, range of motion). Moreover, some measured traits are modifiable by the self or through intervention. Thus, while we consider traits *relatively* invariant characteristics of individuals, they are not entirely static and can be expected to change over time necessitating recurring measurement (perhaps annually or biannually). While we made all attempts to execute study events within a few weeks of baselining to reduce variance in trait measures, this could indeed be a source of variance in our results. Indeed, more timely baselining (e.g., same day or week) and the measurement of ongoing states that influence performance [e.g., sleep, stress, hydration; (Brunyé et al., 2021)] will likely reduce model error and increase the proportion of outcome variance accounted for in our models.

It is worth noting that many feature selection processes are generally agnostic to the directionality of relationships between features and outcomes; indeed, most machine learning and artificial intelligence feature selection approaches (e.g., random forest, neural networks) provide metrics related to feature importance (which can be based on many different factors), but not the directionality of relationships between features and outcomes. At an extreme, the precise nature of these relationships is completely obscured by some feature selection and classification approaches (i.e., the black box phenomenon) (Oh et al., 2019; Liang et al., 2021). We explored the use the elastic net regression to increase transparency, quantify the variance accounted for by selected features, and reveal some insights into the strength and directionality of relationships between features and outcomes. However, apparent counterintuitive results may not hold practical value for understanding mechanistic relationships between features and outcomes; rather, they should motivate continuing research. Furthermore, while many of the identified features had small but non-zero coefficients characterizing their relationships to performance outcomes, a combination of many features with small effects can collectively have a substantial impact. It is also worth noting that due to the regularization techniques used to reduce model complexity, many feature coefficients have been shrunken toward zero. Although this does help to increase the generalizability of the model, it also means the true relationships between the feature and outcome may not be fully captured by the model. Overall, it is still meaningful when a feature is selected as a relevant predictor. The overall predictive power of the model might rely on the combined contributions of many such small-effect features; also, the regularization technique used herein ensured that these features contribute optimally without making the model overly complex or sensitive to noise.

## 5 Conclusion

In conclusion, predicting human performance outcomes is a challenging endeavor, and even highly comprehensive baseline assessment batteries only appear to account for low-to-moderate amounts of variance in applied performance outcomes. Complementing these relatively invariant predictors with an understanding of ongoing personnel states, such as through wearable biosensing, will likely prove valuable for performance prediction. Moving forward, interdisciplinary research efforts integrating psychological, physiological, and operational perspectives will be instrumental in advancing our understanding of human performance optimization in dynamic and challenging environments.

## Data Availability

The data analyzed in this study is subject to the following licenses/restrictions: Data were collected on a specialized and sensitive military population. Contact the corresponding author for data inquiries. Requests to access these datasets should be directed to Tad Brunye, thaddeus.t.brunye.civ@army.mil.
